# Cost-effectiveness analysis for midostaurin versus standard of care in acute myeloid leukemia in the United Kingdom

**DOI:** 10.1186/s12962-018-0153-4

**Published:** 2018-10-04

**Authors:** Gabriel Tremblay, Mike Dolph, Sachin Patel, Patricia Brandt, Anna Forsythe

**Affiliations:** 1 Purple Squirrel Economics, 4 Lexington Avenue, Suite 15K, New York, NY 10010 USA; 2 Novartis Pharmaceuticals UK Limited, Frimley Business Park, Frimley, Camberley, Surrey GU16 7SR UK; 3 Novartis Pharmaceuticals, 1 Health Plaza, East Hanover, NJ 07936 USA

**Keywords:** Acute myeloid leukemia, Fms-like tyrosine kinase 3, Cost-effectiveness analysis, Life years, Quality-adjusted life years, Incremental cost-effectiveness ratio

## Abstract

**Aims:**

Midostaurin (MIDO) has been proposed for the treatment of newly-diagnosed adult patients with FMS-like tyrosine kinase 3 mutation-positive (FLT3+) acute myeloid leukemia (AML) in combination with standard chemotherapy. The cost-effectiveness of MIDO and standard of care (SOC) followed by MIDO monotherapy was compared to SOC alone for newly-diagnosed FLT3+ AML in the UK.

**Methods:**

A partitioned survival model was developed from a UK public healthcare system perspective to compare the cost-effectiveness of MIDO plus SOC and SOC over a lifetime horizon. The model included the following health states/partitions: induction, consolidation, monotherapy, complete remission (CR), relapse, stem cell transplantation (SCT), SCT recovery, and post-SCT recovery. Data on CR, overall survival, and adverse events were obtained from a Phase III clinical trial. Overall survival was extrapolated beyond the trial horizon using a ‘cure model’ approach and data from the Office for National Statistics. Utilities were identified via a systematic review. Routine care utilization was obtained from the National Institute for Health and Care Excellence single technology appraisal for azacitidine in AML (TA399). The costs of drugs and administration, adverse events, hospitalizations, physician visits, and end-of-life care were incorporated.

**Results:**

Incremental life years (LYs) and quality-adjusted life years (QALYs) gained by patients on MIDO and SOC versus SOC were 1.67 and 1.47, respectively. At an incremental cost of £54,072 over a lifetime horizon, the ICER was £32,465 per LY and £36,826 per QALY. Sensitivity analyses were generally consistent with the base case findings.

**Conclusions:**

With limited treatments in FLT3+ AML, MIDO represents a clinically significant advance in the management of newly-diagnosed AML. Using a threshold of £50,000 per QALY for end-of-life treatment, MIDO was shown to be a cost-effective option for newly-diagnosed FLT3+ AML.

**Electronic supplementary material:**

The online version of this article (10.1186/s12962-018-0153-4) contains supplementary material, which is available to authorized users.

## Background

Acute myeloid leukemia (AML) is the most common acute adult leukemia, with the lowest survival rates. As its progression is aggressive and treatment must begin immediately following diagnosis, AML is considered a medical emergency [1]. Estimates of 5-year survival range from 12 to 27% overall [2, 3]. Mutation of fms-like tyrosine kinase 3 (FLT3) is present in around 30% of all AML and confers an even poorer prognosis, with a median overall survival (OS) of less than 12 months with current standard of care (SOC) therapy [4, 5]. These patients are considered high-risk, their therapeutic options are limited and recurrence rates are high.

Despite a continuous research effort, there have been no significant advances in the management of AML in recent decades [1]. Intensive induction chemotherapy is recommended for patients fit enough to tolerate such a regime. Induction therapy, composed of an anthracycline agent and cytarabine, is followed by consolidation chemotherapy, typically consisting of high-dose cytarabine, in patients who achieve complete remission (CR). Patients may also receive post-remission maintenance therapy if it is deemed appropriate. Many patients ultimately undergo stem cell transplantation (SCT), including a large proportion of those considered intermediate- or high-risk [6]. Patients with FLT3 mutation-positive AML are often considered for SCT. However, SCT itself is associated with significant morbidity and mortality, primarily due to chronic graft-versus-host disease, which can affect up to 80% of transplant recipients [7, 8]. There is, thus, a need for better, safer treatments for FLT3 mutation-positive and other high-risk AML patients.

Midostaurin (MIDO) is the first targeted therapy to significantly improve OS in newly diagnosed FLT3 mutation-positive AML patients. Administered in combination with chemotherapy during induction and consolidation and subsequently as a monotherapy during maintenance in the RATIFY international phase III trial of 717 patients age 18–59, MIDO significantly improved OS as compared to placebo: an increase from 25.6 to 74.7 months. Event-free survival, disease-free survival and remission duration were also prolonged. Median follow-up was 60.2 months for both groups [9, 10]. Importantly, the addition of MIDO to SOC was shown to improve survival without compromising safety, as there were not significant differences with the incidence of grades 3/4 or severe adverse events between the arms [9]. Midostaurin is orally administered and generally well tolerated: results from the RATIFY trial suggest that it could enhance the SOC and improve therapeutic outcomes in AML while favoring patient convenience.

Following priority review, MIDO was approved by the US Food and Drug Administration for the treatment of newly diagnosed FLT3 mutation-positive AML in 2017 [11–13]. European Medicines Agency approval followed, and it appears increasingly likely that MIDO will soon become intrinsic to FLT3 mutation-positive AML treatment strategies [12, 14]. In this study, we begin the important process of estimating the costs and outcomes associated with incorporating MIDO in a wider context in the UK.

## Methods

### Model perspective and patient populations

A cost-effectiveness model was developed according to international good research practices for modelling and methods guidance published by the National Institute for Health and Care Excellence (NICE) [15]. A partitioned survival model was implemented from the perspective of the National Health Service (NHS) and personal and social services in England and Wales and a lifetime horizon beginning at treatment initiation. The cycle length was 28 days, as treatment cycles lasted 28 days in the RATIFY clinical trial.

The study population was composed of previously untreated adult AML patients with mutated FLT3 who were eligible to receive standard induction and consolidation chemotherapy, corresponding to the intention-to-treat population of the RATIFY clinical trial and the intended use for MIDO therapy. These patients received MIDO and standard chemotherapy or placebo and standard chemotherapy. The model presents estimates of life years (LY) and quality-adjusted life years (QALY) that would be gained through the incorporation of MIDO treatment. The model was created using Microsoft Excel and the survival analyses were performed in Stata 14.

### Health states and treatments

Model health states, or partitions, included: AML induction/diagnosis (initial state), CR, relapse, and SCT (including SCT treatment, SCT recovery, and post-SCT recovery). These health states were selected based on the clinical pathway and current guidelines for newly diagnosed FLT3 mutation-positive AML.

At the initiation of primary therapy, patients entered the induction chemotherapy state and received either MIDO (days 8–21) in addition to SOC (composed of cytarabine (days 1–7) and daunorubicin (days 1–3)), or SOC alone.

**Fig. 1:**
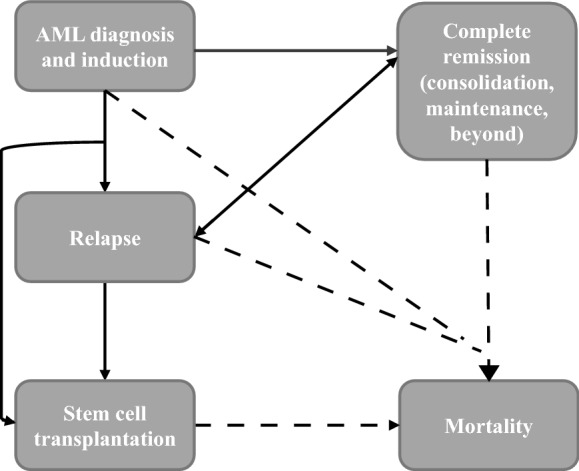
Model framework. *AML* acute myeloid leukemia, dashed lines represent transitions to mortality

Patients left the induction state if they achieved CR and became eligible for consolidation therapy (consisting of high dose cytarabine and MIDO), if they relapsed, or if they started SCT. In RATIFY, patients who maintained remission following consolidation received MIDO. Figure 1 depicts possible health state transitions. The model framework was based on the typical AML treatment pathway and previously published economic studies in AML [16, 17].

Treatment duration, including all phases, was based on the proportion of patients on treatment in RATIFY. The estimation of patients occupying each health state was derived directly from the clinical outcomes of the RATIFY trial.

### Efficacy measures and survival extrapolation

In the RATIFY Phase III trial, MIDO treatment produced promising results. Median OS for patients who received MIDO was 74.7 months versus 25.6 months for patients who received placebo, representing a risk reduction of 23%. As such, MIDO was the first therapy demonstrated to significantly prolong OS in recent decades. Event-free survival (EFS) also favored MIDO with a median event-free survival period of 8 months versus 3.6 for placebo, a 21% reduction in risk.

#### Overall survival

**Fig. 2:**
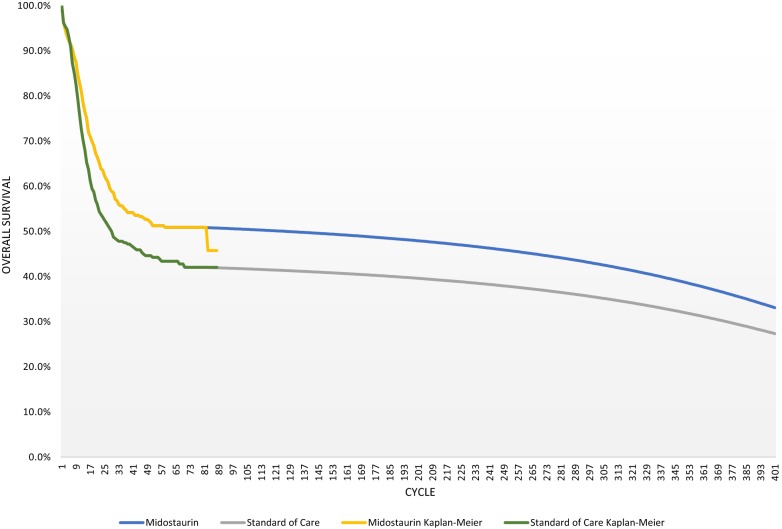
Overall survival cure model long term extrapolation

Figure 2 presents the long-term extrapolation of OS. In the model, data from the RATIFY randomized controlled trial for OS was extrapolated beyond the trial horizon using a cure model approach, which assumes subsequent natural mortality, based on 2013–2015 data from the Office for National Statistics [18]. Clinical experts indicated that patients alive at the end of the trial would typically experience a rate of death equal to that of the general population after 3 years. Gains in OS were expected to be maintained and the survival plateau observed at the end of the trial was expected to persist. Over a lifetime, the gain in OS observed during the trial for MIDO versus SOC is expected to be maintained. The resulting curve is consistent with key opinion leader interviews, while extrapolations produced using other techniques were not.

A range of extrapolation approaches were considered (Gamma, Log Logistic, Weibull, Exponential) for both OS and EFS before a cure model was selected as the most reasonable fit, as clinical experts considered that none of the single parametric functions (or parametric function in addition to the Kaplan–Meier) examined provided a reasonable extrapolation and that patients still alive by the time the trial ended would typically experience the same rate of death as the general population. While the cure model was used, the impact of using other extrapolation forms were explored in scenario analysis (presented with “Results”).

In the base case model, the cure model is fitted from the last event. It should be noted, however, that for the MIDO arm, the Kaplan–Meier quickly dropped at cycle 81 due to one event, which was not observed in the SOC arm. It is unclear whether this is due to an inconsistency in the data, and therefore the cure-model has been fitted prior to that event in our base case for MIDO. It should be noted that fitting the cure model from the last event could be an equally plausible scenario and therefore results after fitting the extrapolation from the last measurement are presented in scenario analysis.

#### Event-free survival

In addition to OS and EFS, the RATIFY clinical trial provided data on CR, SCT, and adverse events. Event-free survival was used as a secondary endpoint, patients reached EFS if they did not achieve remission in the first two cycles, relapsed, discontinued therapy, entered stem cell therapy, or died. Oncology trials traditionally use progression-free survival, the time from randomization to death from any cause, to estimate progression and relapse. Since EFS is a poor proxy for PFS in the estimation of relapse, CR and SCT patient level data were directly used to estimate the proportion of patients in these health states.

The proportion of patients in CR after trial cut-off was extrapolated based on the EFS extrapolation (i.e., the same rate of EFS decay was used for CR decay), ensuring the CR curve was internally consistent with the EFS curve.

#### Stem cell therapy

Overall, 57% of patients received SCT in the RATIFY trial and this proportion was similar between treatment groups (59.4% in the MIDO arm and 55.2% in the SOC arm). The slightly higher rate of SCT in the MIDO arm may be attributed to the higher CR rate seen in this group, which ultimately lead to the higher overall SCT cost in the MIDO group.

Following trial cut-off, SCT uptake rate was carried forward from the last trial measurement. A clinical expert noted that among patients who survive longer than 6 years, expected survival is similar for patients who received SCT and those who did not. Therefore, the SCT survival beyond the trial-off was based on OS trends: patients who received SCT were assumed to reach mortality at the same rate as the overall surviving population.

### Costs

Across each health state, the model incorporated the following costs: drugs and administration, treatment for adverse events, and medical costs for hospitalizations, physician visits, and end-of-life care. As per NICE guidelines, costs and utilities were discounted at a rate of 3.5% per year. Any cost inputs that were based on data prior to the model year were inflation-adjusted at 3.1% [15].

#### Drug costs

Utilization of primary therapy was based on patient-level data from the RATIFY trial. Drug acquisition costs for chemotherapy were from the British National Formulary, while the manufacturer provided pricing for MIDO. The drug costs for MIDO include wastage, which rounds fractions of doses up to the nearest whole value. No administration costs were assumed for MIDO, as it is an oral therapy. Treatment durations were derived from the proportion of patients in each cycle in RATIFY. Doses were based on a mean body surface area of 1.90 m^2^ (based on RATIFY clinical trial patient-level data), and dose reduction was calculated using the within-trial doses received. Primary therapy costs are summarized in Table 1.

**Table 1:** Efficacy data summary (from the RATIFY clinical trial cut-off)

	MIDO-treated patients	SOC-treated patients
Overall survival (non-censored), %	46	42
Event-free survival, %	27	18
Stem cell therapy received during trial, %	59	55

Secondary therapy (fludarabine, cytarabine, idarubin, filgrastim, FLAG-IDA) utilization and duration were based on Kantar Health estimates [20]. Cost per cycle of secondary therapy was calculated in the same way as primary therapy, though without dose reduction. Data on the market share uptake of these treatments was based on a survey of 50 UK physicians [21].

#### Routine care costs

Routine care unit costs were obtained from various sources such as the Personal Social Services Research Unit (PSSRU) Unit Costs of Health and Social Care, inflation-adjusted to 2017 values [22] (Table 2).

**Table 2:** Reference costs for routine medical care

Health states	Unit cost (£)	Cost per minute (£)	Source
CNS haematologist	81	1.35	PSSRU, 2015—(1 h client contact) 10.7 advanced nurse (includes lead specialist, clinical nurse specialist, senior specialist)
Consultant	105	1.75	PSSRU, 2015—(1 h client contact) 115.5 consultant (medical)
Day care nurse	44	0.73	PSSRU, 2015—10.6 nurse GP practice (per hour)
Day care specialist registrar	41	0.68	PSSRU, 2015—1 h cost—15.3 registrar group
District nurse	65	1.08	PSSRU, 2015—10.4 nurse specialist (community) (per hour patient related work)
Doctor	101	1.68	PSSRU, 2015—15.4 associate specialist (1 h)
Junior doctor	30	0.50	PSSRU, 2015—15.2 foundation house officer 2 (per hour)
Pharmacist	63	1.05	PSSRU, 2015—13.6 hospital pharmacists (patient related activity)—1 h
Oncology nurse	81	1.35	PSSRU, 2015—10.7 advanced nurse (includes lead specialist, clinical nurse specialist, senior specialist) per hour
Inpatient day	631	0.44	SA25F, acute myeloid leukaemia without CC (combined day case/ordinary elective spell tariff (£))
FLT3-ITD testing	150		KOL assumption

A systematic review of the literature was conducted to identify studies which reported resource utilization that could be used within the economic model. Although a number of studies were identified, specific detail on the numbers used for utilization were lacking. Therefore, in the absence of quality data, resource utilization (with the exception of stem cell transplant) in the model are based on resource utilization reported in NICE TA399 for azacitidine [23]. In brief, healthcare resource use in NICE TA399 were estimated based on a clinician survey conducted by the company amongst 7 clinicians with the average of responses used in the model.

Healthcare resource utilizations were estimated for four health states; (1) induction/pre-response, (2) in remission (CR), (3) not in remission (which could include partial response, stable disease or not in remission without progressive disease) and (4) progressive disease. Healthcare resource utilizations were also estimated separately by treatment arms in people initiating azacitidine and people initiating conventional chemotherapy regimens. (Specific routine care utilization rates are presented in Additional file 1: Appendix.

#### Stem cell transplantation costs

Average costs associated with SCT, as presented in Table 3, were obtained from NHS Reference Costs and included the costs of SCT, peripheral blood stem cell harvest costs, hospitalization costs, and medical oncologist follow-up costs. The average cost of SCT used in the model was £25,116 (a detailed breakdown of this cost is shown in Additional file 1: Appendix).

**Table 3:** Drug dosages and costs (primary therapy)

Phase	Arm	Regimen	Dose	Mg per cycle^a^	Vial size mg (or tablet)	Price per vial/tablet, £	Cost per cycle as per indication, £	Cost with wastage and dose reduction, £
Induction	MIDO	CYTA	200 mg/m^2^/day (1–7)	2660	500	£19.50	£103.74	£113.59
		DAUNO	60 mg/m^2^/day (1–3)	342	20	£65.00	£1111.50	£1216.61
		MIDO	50 mg (2 × 25) twice per day (8–21)	1400	25	£100.18	£6024.10	£5572.98
		Total cost per cycle					***£7239.34***	***£6903.18***
	SOC	CYTA	200 mg/m^2^/day (1–7)	2660	500	£19.50	£103.74	£112.68
		DAUNO	60 mg/m^2^/day (1–3)	342	20	£65.00	£1111.50	£1216.16
		Total cost per cycle					*£1215.24*	*£1328.84*
Consolidation	MIDO	High-dose CYTA	3000 mg/m^2^/day (1, 3, 5) twice/day	34,200	500	£19.50	£1333.80	£1590.36
		MIDO	50 mg (2 × 25) twice/day (8–21)	1400	25	£100.18	£6024.10	£5753.67
		Total cost per cycle					***£7357.90***	***£7344.03***
	SOC	High-dose CYTA	3000 mg/m^2^/day (1, 3, 5) twice/day	34,200	500	£19.50	£1333.80	£1585.32
		Total cost per cycle					*£1333.80*	*£1585.32*
Monotherapy	MIDO arm	MIDO	50 mg (2 × 25) twice/day (1–28)	2800	25	£100.18	£12,048.19	£11,596.39
		Total cost per cycle					***£12,048.19***	***£11,596.39***

Cost source: British National Formulary and Novartis

Bold italic indicates total costs, underline indicates values belonging to the MIDO arm in each section

*MIDO* midostaurin, *SOC* standard of care, *CYTA* cytarabine, *DAUNO* daunomycin

^a^Mg per cycle based on a body surface area of 1.9 m^2^ (from the RATIFY clinical trial)

#### Adverse event costs

The prevalence of adverse events is typically high in AML: in RATIFY, 99% of patients experienced at least one grade 3/4 adverse event. Adverse events with a prevalence of ≥ 5% in any treatment phase were included in the model. Prevalence was derived from the clinical trial results and specified for each treatment phase, as rates are very different from one treatment phase to the next. Table 4 presents relevant adverse events, their costs, and their prevalence in each treatment population. Costs were obtained from the NHS National Prices and National Tariff Workbook [24].

**Table 4:** Adverse event cost and prevalence

Adverse event	Unit cost (£)	Induction		Consolidation		Monotherapy	
		MIDO (%)	SOC (%)	MIDO (%)	SOC (%)	MIDO (%)	SOC (%)
Platelet count decreased	£2469.87	50.15	49.99	33.07	34.16	0.18	1.71
Neutrophil count	£1076.37	48.03	49.20	32.33	34.50	0.89	1.05
Hemoglobin	£1142.90	45.90	42.55	28.75	28.69	0.09	0.00
Febrile neutropenia	£3579.00	39.06	41.13	21.00	20.49	0.09	0.00
Leukopenia NOS	£1076.37	11.85	13.60	8.04	10.25	0.27	0.00
Lymphopenia	£1956.51	7.14	8.70	6.85	9.05	0.71	0.26
Diarrhea NOS	£817.76	6.38	6.96	1.79	2.22	0.09	0.26
Hypokalemia	£1320.26	5.62	6.80	2.09	3.25	0.00	0.13
Alanine aminotransferase increased	£2421.00	3.19	2.85	3.28	2.22	0.45	0.53
Dermatitis exfoliative NOS	£1057.00	6.38	3.48	0.89	0.68	0.09	0.00
Fatigue	£664.00	2.13	3.64	2.83	1.71	0.09	0.13
Hyperglycemia NOS	£1053.56	1.52	1.90	1.79	1.37	0.27	0.66
Pneumonitis NOS	£892.00	2.74	2.85	1.19	1.71	0.00	0.13
Nausea	£664.00	2.43	4.27	0.74	1.71	0.00	0.00
Hyponatremia	£959.00	4.10	2.85	0.74	0.68	0.00	0.00
Blood bilirubin increased	£959.00	3.34	2.85	0.60	0.68	0.00	0.00
Infection	£3630.12	1.37	1.27	1.94	1.37	0.09	0.00
Hypophosphatasemia	£959.00	2.28	3.64	0.74	0.85	0.00	0.26
Gamma-glutamyltransferase increased	£959.00	1.67	2.85	0.74	1.02	0.09	0.00
Hypocalcemia	£959.00	2.74	2.53	0.30	0.34	0.00	0.00
Radiation mucositis	£664.00	2.28	3.32	0.30	1.02	0.00	0.00
Hypoalbuminemia	£959.00	2.58	2.69	0.45	0.17	0.00	0.00
Syncope	£664.00	0.46	0.00	1.79	1.20	0.00	0.13
Total cost, £		4515.62	4624.45	2768.40	2838.50	54.93	87.58

*MIDO* midostaurin, *SOC* standard of care, *NOS* not otherwise specified

#### Mortality costs

Costs obtained from the Nuffield Trust describing mortality included acute hospital care, local authority-funded social care, district nursing care, and general practitioner visits [25]. These costs were summed to obtain the cost per mortality event, and were then adjusted for inflation to 2017 values. In the model, cost per-mortality was £14,887 (a detailed breakdown of this cost is presented in Additional file 1: Appendix).

### Utilities

As no within-trial health-related quality of life (HRQoL) data were collected during the RATIFY trial, utility values were obtained following a systematic review of the literature. The systematic literature review was described in full by Forsythe et al. [26]. For SCT, EuroQol-5 Dimension (EQ-5D) utility values were calculated using QLQ-30 data from Grulke et al. and an algorithm by Crott et al. presented below [27, 28]. The QLQ-30 data included: before SCT, during hospitalization, up to 6 months after SCT, and > 1 year after SCT.

$$\begin{aligned} EQ - 5D \, utility\, = & \,0.85927770 \\ & - \,0.0069693*\left( {Physical \, Functioning} \right)\, - \,0.0087346*\left( {Emotional \, Functioning} \right) \\ & \, - \,0.0039935*\left( {Social \, Functioning} \right)\, + \,0.0000355*\left( {Physical \, Functioning} \right)^{2} \\ & \, + \,0.0000552*\left( {Emotional \, Functioning} \right)^{2} \, + \,0.0000290*\left( {Social \, Functioning} \right)^{2} \\ & \, + \,0.0011453*\left( {Constipation} \right)\, + \,0.0039889*\left( {Diarrhea} \right)\, + \,0.0035614*\left( {Pain} \right)\, \\ & - \,0.0003678*\left( {Sleep} \right)\, - \,0.0000540*\left( {Diarrhea} \right)^{2} \, + \,0.0000117*\left( {Sleep} \right)^{2} \\ \end{aligned}$$

These state-specific utility values were applied to the proportion of patients in the state to estimate HRQoL. The utility values were assumed to incorporate disutility related to toxicity and adverse events resulting from treatment. Table 5 presents the health state utilities used in the model and their sources.

**Table 5:** Health state utilities used in the model

Utility state	Utility values used in base case (literature)	Values used in scenario analysis (TTO)	Reference (literature values)	Data source
Induction treatment^a^	0.648	0.162	Uyl-de Groot et al. 1998 [18]	Measured EQ-5D
Consolidation treatment^a^	0.710	0.568	Batty et al. 2014 [19]	Calculated from published literature
Monotherapy treatment^a^	0.810	0.889	Batty et al. 2014 [19]	Calculated from published literature
Complete remission	0.830	0.887	Leunis et al. 2014 [29]	Measured EQ-5D
Relapse	0.530	0.505	Pan et al. 2010 [30]	Mapped from QLQ-C30
SCT treatment^a^	0.613	− 0.210	Source for algorithm—Crott et al. 2010 [27]; Source of QLQC30 data—Grulke et al. 2012 [28]	Mapped from QLQ-C30
SCT recovery	0.810	0.748	Source for algorithm—Crott et al. 2010 [27]; Source of QLQC30 data—Grulke et al. 2012 [28]	Mapped from QLQ-C30
Post-SCT recovery	0.826	0.715	Source for algorithm—Crott et al. 2010 [27]; Source of QLQC30 data—Grulke et al. 2012 [28]	Mapped from QLQ-C30

*TTO* time trade-off, *STC* stem cell transplantation, *QLQC30* Quality of Life Questionnaire Core 30 (produced by the European Organization for Research and Treatment of Cancer)

^a^Includes treatment disutility

### Sensitivity analyses

Probabilistic sensitivity analysis was conducted to evaluate the uncertainty of the results. Probabilistic distributions were applied to the base case model following standard statistical methods, in which beta distributions are assigned to binomial data, gamma or log-normal to right skew parameters, log-normal to relative risk or hazard ratios, and logistic to odds ratios [31].

A log-normal distribution was applied to efficacy parameters (OS and SCT rate) with standard error based on the trial. Dosing parameters were divided between log-normal distributions with standard error based on the trial (body surface area, kilograms) and beta distributions (treatment duration partition and dosing intensity per phase) with standard error based on (sqrt((p*(1 − p)/n)))^2^. The distribution of cost in this analysis appeared to be right-skewed and therefore log-normal distributions were applied for all the cost variables (standard error was set at ± 20%). Gamma distributions were used for the utility parameters and standard error was ± 10% based on a literature review. The point estimates and variations used are presented in Additional file 1: Appendix. Probabilistic sensitivity analysis results are summarized using a cost-effectiveness plane and a net benefit analysis (presented in “Results” section).

Deterministic sensitivity analysis was used to assess variations of model parameters that had the greatest effect on incremental cost effectiveness ratios (ICER) generated by the cost-effectiveness analysis.

Furthermore, additional scenario analysis was conducted in order to assess the impact of major changes to model assumptions.

## Results

### Overall costs

Costs, broken down by category, are summarized in Table 6. The overall cost per arm was estimated at £267,325 for MIDO therapy, and £213,253 for SOC.

**Table 6:** Summary of predicted resource use

Costs	MIDO	SOC	Difference
Drug costs			
Induction and initiation	£8242	£1747	£6495
Consolidation	£13,790	£2602	£11,188
Monotherapy	£36,021	£0	£36,021
Secondary therapy costs	£1354	£1531	− £177
Adverse events costs			
AEs induction costs	£5293	£5557	− £264
AEs consolidation costs	£5198	£4659	£539
AEs monotherapy costs	£171	£0	£171
Routine care costs			
Routine care costs during drug treatment	£9586	£8045	£1541
Routine care after drug treatment	£122,768	£132,429	− £9661
SCT cost	£55,245	£46,172	£9073
Mortality costs	£9656	£10,511	− £855
Total	£267,325	£213,253	£54,072

*MIDO* midostaurin, *SOC* standard of care, *AE* adverse event, *SCT* stem cell transplantation

### Cost-effectiveness

The partition survival model developed in this analysis was used to estimate the outcomes and costs of adding MIDO to SOC AML treatment. Over a lifetime horizon, MIDO treatment was associated with 10.60 LYs saved and 7.79 QALYs saved versus 8.93 LYs and 6.32 QALYs for the SOC alone. The LYs and QALYs gained by patients on MIDO and SOC versus the SOC alone were therefore 1.67 and 1.47, respectively. At an incremental cost of £54,072 over a lifetime horizon, the ICER per LY was £32,465 and £36,826 per QALY.

### Probabilistic sensitivity analysis

**Fig. 3:**
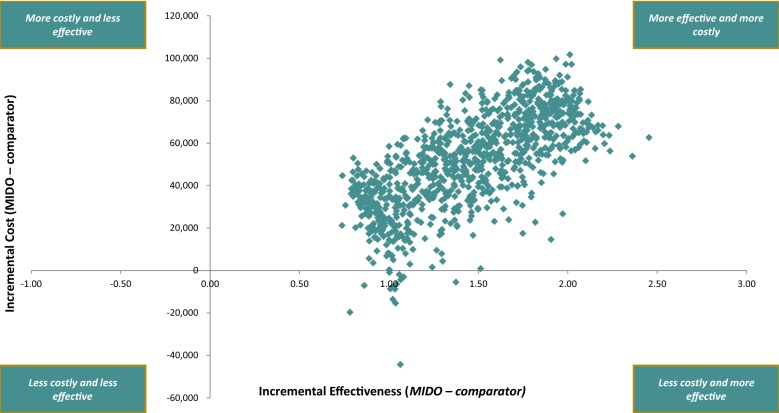
Cost-effectiveness plane

Figure 3 presents the results of the probabilistic sensitivity analysis. Based on this analysis, the average number of QALY gained with MIDO therapy compared to SOC was 1.46 (95% CI 0.89, 2.04). The average incremental cost was £51,621 (95% CI £15,710, £84,810), resulting in an average ICER of £35,435 (95% CI £14,296, £52,641). The probabilities of MIDO cost-effectiveness at thresholds of £30,000 and £50,000 were 39.2% and 97.3%, respectively.

**Fig. 4:**
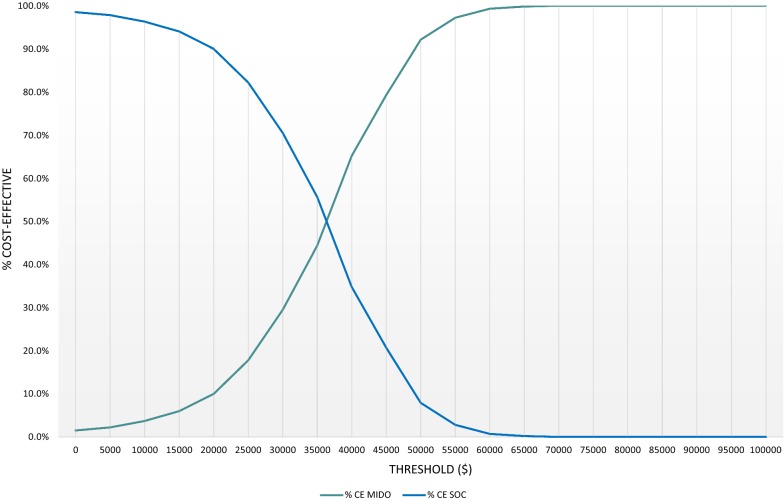
Net benefit threshold: midostaurin versus standard of care. *CE MIDO* cost-effectiveness of midostaurin, *CE SOC* cost-effectiveness of standard of care

Figure 4 presents the net benefit threshold for MIDO compared to SOC, which shows the probability of the net benefit of MIDO being greater than zero (teal line) across a cost-effectiveness threshold spectrum.

### Deterministic sensitivity analysis

**Fig. 5:**
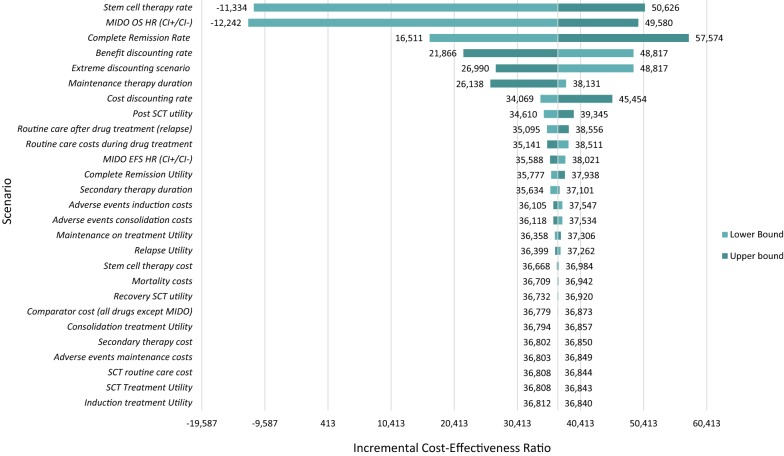
Deterministic sensitivity analysis results (incremental cost-effectiveness ratio per quality-adjusted life year). *SCT* stem cell transplantation, *MIDO* midostaurin, *OS* overall survival, *HR* hazard ratio, *CI* confidence interval, *EFS* event-free survival

Figure 5 depicts the results of the deterministic sensitivity analysis. The deterministic sensitivity analysis was relatively consistent with the base case findings. Many of the deterministic changes that were applied in sensitivity analyses did not lead to significant changes in the incremental cost-effectiveness ratios and suggest the conclusions from the base case model are robust to varying individual parameters. Of all variations assessed, the analysis was most sensitive to variations in stem cell therapy rate, MIDO therapy OS, differences in CR rate, and discounting rates.

### Additional scenario analysis

**Fig. 6:**
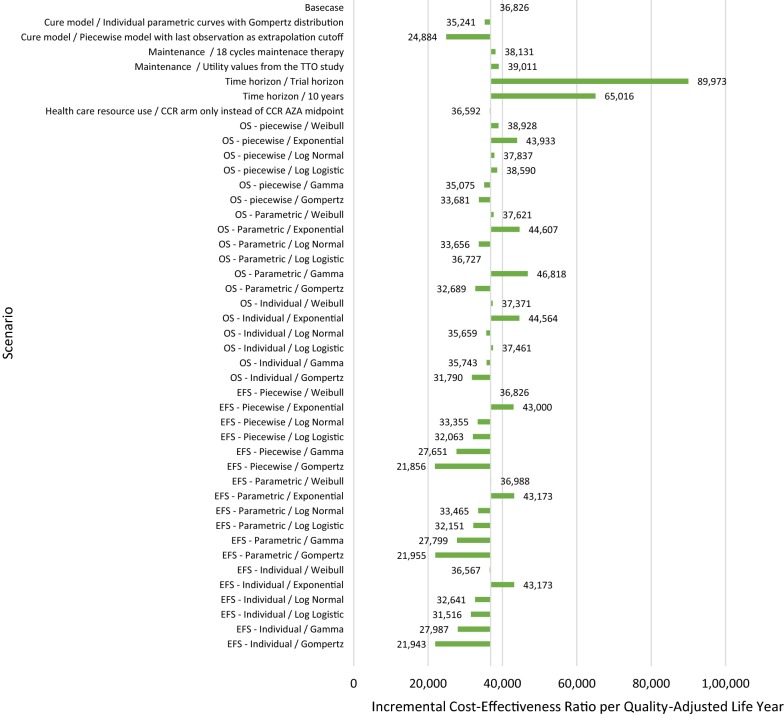
Additional scenario analysis results (incremental cost-effectiveness ratio per quality-adjusted life year). *OS* overall survival, *EFS* event-free survival, *CCR* conventional chemotherapy regimen, *AZA* azacitidine

Figure 6 presents the results of the additional scenario analysis, which was relatively consistent with the base case findings, but showed most sensitivity to changes in time horizon.

## Discussion

AML progresses aggressively, particularly in the 30% of patients who are FLT3 mutation-positive. Following promising phase III trial results and accompanying its clinical implementation, the cost-effectiveness of MIDO for the treatment of FLT3 mutation-positive AML was methodically analysed, as dictated by the standards of the field.

The economic evaluation, conducted from a UK healthcare system perspective, expanded on data from RATIFY and incorporated costs from local data sources. OS with or without the addition of MIDO to the SOC was extrapolated using a cure model, as validated by clinical experts. Estimated using a partitioned survival model, patients in the MIDO arm gained 1.67 LYs and 1.47 QALYs versus SOC alone. Over a lifetime horizon at an incremental cost of £54,072, the ICER was £32,465 per LY and £36,826 per QALY. MIDO, therefore, meets NICE end-of-life criteria and may be considered a cost-effective treatment for newly diagnosed patients with FLT3 mutation-positive AML. As such, this model supports the recommendation and reimbursement of MIDO in the indicated population.

Sensitivity analyses were also performed and supported these conclusions. Probabilistic sensitivity analysis demonstrated that MIDO in combination with chemotherapy had an average ICER of £35,435 per QALY versus standard-of-care, with a 39.2% probability of being cost-effective at a threshold of £30,000 per QALY and a 97.3% probability at a threshold of £50,000 per QALY. Deterministic sensitivity analysis showed greatest sensitivity to changes in SCT rate and MIDO OS relative to SOC. Lastly, additional scenarios showed greatest sensitivity to changes in the model horizon.

The model, though comprehensive, had some limitations. Where specific data were unavailable, as for market share and the duration of SCT and recovery, estimates were obtained from interviews with clinical experts. Fifty experts were interviewed, a larger sample could provide further insights, particularly regarding market share. Additionally, the model relied on utility values obtained from the literature and mapped to the health states: a limitation that was unavoidable, as these data were not collected during the RATIFY clinical trial. Lastly, patients in the SCT health state could only transition out through mortality, dictating necessarily that no relapse or subsequent therapy occurred after SCT.

## Conclusions

In summary, MIDO is considered a breakthrough in the management of FLT3 mutation-positive AML, a product of 30 years’ research effort. Our analysis demonstrates that this necessary improvement to the SOC treatment for AML in this patient population would also be cost-effective and thus may serve as an important contribution to the existing literature.

## Additional file

**Additional file 1.** Appendix.

## Abbreviations

AML: acute myeloid leukemia
FLT3: FMS-like tyrosine kinase 3
SOC: standard of care
SCT: stem cell transplantation
MIDO: midostaurin
FLAG-IDA: fludarabine, cytarabine, granulocyte-colony stimulating factor, idarubicin
EQ-5D: EuroQol-5 Dimension
QLQ-30: quality of life questionnaire core 30
CI: confidence interval
NICE: National Institute for Health and Care Excellence
NHS: National Health Service
LY: life year
QALY: quality-adjusted life year
CR: complete remission
HRQoL: health-related quality of life
ICER: incremental cost-effectiveness ratio
OS: overall survival
CE: cost-effectiveness
HR: hazard ratio
EFS: event-free survival
NOS: not otherwise specified
