# Supplementary material for: Cost-effectiveness analysis for midostaurin versus standard of care in acute myeloid leukemia in the United Kingdom
Source: Cost Eff Resour Alloc. 2018 Oct 4;16:33. doi: 10.1186/s12962-018-0153-4 (PMC6172753; doi:10.1186/s12962-018-0153-4)
Supplement: Supplementary file 1 — Additional file 1. Appendix. [file 12962_2018_153_MOESM1_ESM.docx]

**Appendix**

Table S1. National Health Service reference costs for stem cell transplantation

| **Code** | **Description** | **Number of interventions (used in the weighted average0** | **Cost, £** |
| --- | --- | --- | --- |
| SA26A | Peripheral Blood Stem Cell Transplant, Autologous, 19 years and over | 1,877 | 17,344 |
| SA26B | Peripheral Blood Stem Cell Transplant, Autologous, 18 years and under | 161 | 28,980 |
| SA27A | Peripheral Blood Stem Cell Transplant, Syngeneic, 19 years and over | 7 | 18,300 |
| SA27B | Peripheral Blood Stem Cell Transplant, Syngeneic, 18 years and under | 6 | 426 |
| SA38A | Peripheral Blood Stem Cell Transplant, Allogeneic (Sibling), 19 years and over | 204 | 28,176 |
| SA38B | Peripheral Blood Stem Cell Transplant, Allogeneic (Sibling), 18 years and under | 35 | 81,622 |
| SA39A | Peripheral Blood Stem Cell Transplant, Allogeneic (Volunteer Unrelated Donor), 19 years and over | 379 | 33,486 |
| SA39B | Peripheral Blood Stem Cell Transplant, Allogeneic (Volunteer Unrelated Donor), 18 years and under | 34 | 70,445 |
| SA40Z | Peripheral Blood Stem Cell Transplant, Allogeneic (Donor Type Not Specified) | 518 | 38,336 |
|  |  | **Weighted average SCT cost*** | **25,116** |

*SCT: stem cell transplantation*

**Average based on a weighted mean using the number of interventions for each code.*

**Table S2. Health care utilization used in the model (minutes per cycle)**

| **Health states** | **Initiation** | **Induction** | **Second induction and secondary therapy** | **Consolidation** | **Monotherapy/Complete remission** | **Relapse** | **SCT treatment** | **SCT recovery** |
| --- | --- | --- | --- | --- | --- | --- | --- | --- |
| CNS Haematologist |  | 66 | 66 | 33 | 33 | 81 | 0 | 0 |
| Consultant |  | 62 | 62 | 17 | 17 | 36 | 0 | 0 |
| Day care nurse |  | 116 | 116 | 13 | 13 | 138 | 0 | 0 |
| Day care SpR |  | 68 | 68 | 28 | 28 | 54 | 0 | 0 |
| District Nurse |  | 42 | 42 | 13 | 13 | 35 | 0 | 0 |
| Doctor |  | 38 | 38 | 17 | 17 | 20 | 101 | 101 |
| Jnr doctor |  | 139 | 139 | 11 | 11 | 66 | 0 | 0 |
| Pharmacist |  | 75 | 75 | 2 | 2 | 24 | 0 | 0 |
| Oncology nurse |  | 16 | 16 | 0 | 0 | 3 | 0 | 0 |
| Inpatient day |  | 12290 | 12290 | 828 | 828 | 5702 | 0 | 0 |
| ITD FLT3 testing | 1 |  |  |  |  |  |  |  |

*CNS: central nervous system, Jnr: junior, ITD FLT3: internal tandem duplication FMS-like tyrosine kinase 3, SCT: stem cell therapy*

**Table S3. Mortality costs**

| **Mortality cost** | **Cost element** | **2013 value in the UK (£)** |
| --- | --- | --- |
| Secondary (acute hospital care) | Cost of all hospital contacts | £5,890 |
|  | Cost of emergency inpatient admissions | £4,071 |
|  | Cost of non-emergency inpatient admissions | £1,360 |
|  | Cost of outpatient visits | £378 |
|  | Cost of A&E visits | £80 |
| Local authority funded social care | Cost of local authority-funded social care | £444 |
| District nursing | Cost of district nursing care | £588 |
| GP contacts | Cost of GP visits | £365 |
| Total in 2013 |  | £13,176 |
| Total used in the model (inflation-adjusted for 2017) | | £14,887 |

*Source: Nuffield Trust 2014*

*UK: United Kingdom, A&E: accident and emergency (department), GP: general practitioner*

**Table S4. Probabilistic sensitivity analysis parameters**

|  | **Point estimate** | | **Standard deviation/Error** | |  | |
| --- | --- | --- | --- | --- | --- | --- |
|  | **Mido** | **SOC** | **Mido** | **SOC** | **Distribution** | **Source** |
| **Efficacy and events** | | | | | | |
| OS | 0.774 |  | 0.083 |  | Log-normal | PE based on model and SE based on restricted mean CPKC412A2301 (patient level data)  Note: The OS HR was varied between -1 and +1 SE to avoid crossing with other curves (e.g. EFS) |
| Event free survival | 0.784 |  | 0.068 |  | Log-normal | PE based on model and SE based on restricted mean CPKC412A2301 (patient level data)  Note: The HR was varied between -1.96 and +1.96 SE to avoid crossing with other curves (e.g. EFS) |
| Complete remission | 1.10 |  | 0.038 |  | Log-normal | PE and SD from CSR CPKC412A2301, p 78  Note: The rate was varied between -1.96 and +1.96 SE to avoid crossing with other curves (e.g. OS) |
| SC rate | 1.08 |  | 0.036 |  | Log-normal | PE and SD from CSR CPKC412A2301, p 78  Note: The rate was varied between -1.96 and +1.96 SE to avoid crossing with other curves (e.g. OS) |
| **Dosing** | | | | | | |
| *Treatment duration partition* | Based on patient level data | | Variable for each time point | | Beta | PE for MIDO and SOC from patient level data for each cycle (probability of been treated) and SE for each cycle i.e. (sqrt((p*(1-p)/n)))^2 |
| *Dose intensity – induction* | 92.5 | 0.020 |  |  | Beta | PE from patient level data; SE based on (sqrt((p*(1-p)/n)))^2 |
| *Dose intensity - Consolidation* | 95.5 | 0.018 |  |  | Beta | CSR CPKC412A2301, p 96; SE based on (sqrt((p*(1-p)/n)))^2 |
| *Dose intensity - Monotherapy* | 96.3 | 0.030 |  |  | Beta | CSR CPKC412A2301, p 97; SE based on (sqrt((p*(1-p)/n)))^2 |
| *Body surface area* | 1.90 | | 0.28 | | Log-normal | CSR CPKC412A2301, p 68; SD based on patient level data |
| *KG* | 70.00 | | 21.31 | | Log-normal | Based on CPKC412A2301 patient level data |
| *Secondary therapy duration* | 1.000 | 1.000 | 1.000 | 1.000 | Log-normal | Assumption |
| **Costs** | | | | | | |
| Adverse event costs | Variable | | +/-20% | | Log-normal | PE Based on micro-costing, SD based on assumptions |
| Secondary therapy costs | 3,101 | | +/-20% | | Log-normal | PE Based on micro-costing, SD based on assumptions |
| On treatment routine care costs | Variable | | +/-20% | | Log-normal | PE Based on micro-costing, SD based on assumptions |
| Off-treatment routine care costs | Variable | | +/-20% | | Log-normal | PE Based on micro-costing, SD based on assumptions |
| SCT costs | 25,116 | | +/-20% | | Log-normal | PE Based on micro-costing, SD based on assumptions |
| **Utility** | | | | | | |
| On-treatment utility | Variable | | +/-10% | | Gamma | Literature review |
| Off-treatment utility | Variable | | +/-10% | | Gamma | Literature review |
| SCT utility | Variable | | +/-10% | | Gamma | Literature review |
